# How to assure the quality of clinical records? A 7-year experience in a large academic hospital

**DOI:** 10.1371/journal.pone.0261018

**Published:** 2021-12-09

**Authors:** Enrico Scarpis, Laura Brunelli, Pierfrancesco Tricarico, Marco Poletto, Angela Panzera, Carla Londero, Luigi Castriotta, Silvio Brusaferro

**Affiliations:** 1 Department of Medicine, University of Udine, Udine, Italy; 2 Health District of Udine, Friuli Centrale Healthcare and University Integrated Trust, ASUFC, Udine, Italy; 3 Accreditation, Clinical Risk Management and Performance Assessment Unit, Friuli Centrale Healthcare and University Integrated Trust, ASUFC, Udine, Italy; 4 Hygiene and Clinical Epidemiology Institute, Friuli Centrale Healthcare and University Integrated Trust, ASUFC, Udine, Italy; Tabriz University of Medical Sciences, ISLAMIC REPUBLIC OF IRAN

## Abstract

**Introduction:**

Clinical record (CR) is the primary tool used by healthcare workers (HCWs) to record clinical information and its completeness can help achieve safer practices. CR is the most appropriate source in order to measure and evaluate the quality of care. In order to achieve a safety climate is fundamental to involve a responsive healthcare workforce thorough peer-review and feedbacks. This study aims to develop a peer-review tool for clinical records quality assurance, presenting the seven-year experience in the evolution of it; secondary aims are to describe the CR completeness and HCWs’ diligence toward recording information in it.

**Methods:**

To assess the completeness of CRs a peer-review tool was developed in a large Academic Hospital of Northern Italy. This tool included measurable items that examined different themes, moments and levels of the clinical process. Data were collected every three months between 2010 and 2016 by appointed and trained HCWs from 42 Units; the hospital Quality Unit was responsible for of processing and validating them. Variations in the proportion of CR completeness were assessed using Cochran-Armitage test for trends.

**Results:**

A total of 9,408 CRs were evaluated. Overall CR completeness improved significantly from 79.6% in 2010 to 86.5% in 2016 (p<0.001). Doctors’ attitude showed a trend similar to the overall completeness, while nurses improved more consistently (p<0.001). Most items exploring themes, moments and levels registered a significant improvement in the early years, then flattened in last years. Results of the validation process were always above the cut-off of 75%.

**Conclusions:**

This peer-review tool enabled the Quality Unit and hospital leadership to obtain a reliable picture of CRs completeness, while involving the HCWs in the quality evaluation. The completeness of CR showed an overall positive and significant trend during these seven years.

## Introduction

Clinical record (CR) is the primary tool used by health professionals for recording patient clinical information [1] It supports delivery of care, clinical decision making and continuity of care [2] primary functions [3], and it enables healthcare organizations to operate in accordance with the international quality standards [3–6] and also with national legal requirements [7, 8] (secondary functions [3]. A complete and accurate CR can help achieve safer practices [2], such as better communication and medication reconciliation, which are common reasons for medical errors worldwide [9–11]. Several authors reported that poor quality of the information in CR may be a predictor of poor quality of care [3, 12–16], and may be associated with higher rates of adverse patient safety events [2, 15]. This is true particularly considering adverse drug events, that may be caused by prescribing error in prescription writing, that involved illegibility, ambiguous abbreviations or lack of important piece of information such as date of prescription, dose or route of administration [17]. These aspects must be analysed through CRs review for quality assurance [18]

To determine whether healthcare is safe, effective, efficient, accessible, patient-centred and equitable, as required by IOM [19] and WHO [20], organizations have to measure the quality of care they provide [9]. The rationale for measuring quality improvement and patient safety is the belief that good performance reflects good-quality practice [21]. CR is one of the most appropriate sources in order to measure and evaluate quality of care [2, 3, 7, 12–15] and the diligence with which information is recorded by professionals in it is an important quality indicator that needs to be evaluated in healthcare organizations [2, 14, 16, 22]. Indeed, better recording of clinical information leads to better communication between healthcare providers and could contribute to better patient outcomes and safer healthcare [2, 23]. Developing a skilled and responsive healthcare workforce is a fundamental step in creating a safety climate within any healthcare system [24, 25] and in improving quality of care, such as clinical record keeping [14, 26]. For this reason, the involvement of healthcare workers (HCWs)—intended as direct-Care Healthcare Workers—in the process of quality assessment and management, as well as their collaborative work is crucial [27, 28]. Peer-review [29], as well as feedbacks [30], are widely used strategies to promote improvements and induce desired changes [31].

The Academic Hospital of Udine is the hub hospital of Friuli-Venezia Giulia (Italy). It consists of 42 inpatient units, it has 40,000 inpatients/year, 1,000,000 outpatient/year and 3,700 HCWs are employed. The Hospital implemented a variety of strategies, as contribution to healthcare quality at meso-level [32], to improve hospital system, promoting a safety climate and safety practices through systematic measurement of processes, such as the completeness of CR. This systematic measurement through a peer-review tool for CR quality assurance should help to demonstrate whether improvement efforts lead to change in the desired direction.

This study aims to develop a peer-review tool for clinical records quality assurance, presenting the seven-year experience in the evolution of it, and to describe the CR completeness. The second purpose is to describe the professional diligence toward recording clinical information in the CR through these seven years.

## Methods

### Tool design

Qualified professionals from each unit, 57 doctors and 44 nurses, were selected as direct-Care Healthcare Workers on a voluntary-basis, taking to account their attitude, interest and professional competence in quality of care and patient safety. Thus, they were formally appointed by chief of their unit. They were then trained at regular intervals by the Quality Unit trough meetings and focus groups concerning the structure of the paper-based CR in use, the specific process of CR quality assessment and the tool actually in use. Some of the professionals changed over the years, mainly due to turnover. For this reason, the training was repeated whenever HCWs in charge of the assessment or the tool itself changed across the years.

An electronic peer-review tool was developed to assess the quality of the paper-based CR. HCWs were completely independent in the peer-review process: this was considered as unobtrusive. The peer-review tool included several items that measure the filling in state of specific sections of the CR. Every item was assigned a score of 1 if the specific section was filled in, 0 if it was not, NA (Not Applicable) where the specific item was not applicable.

**Fig 1:**
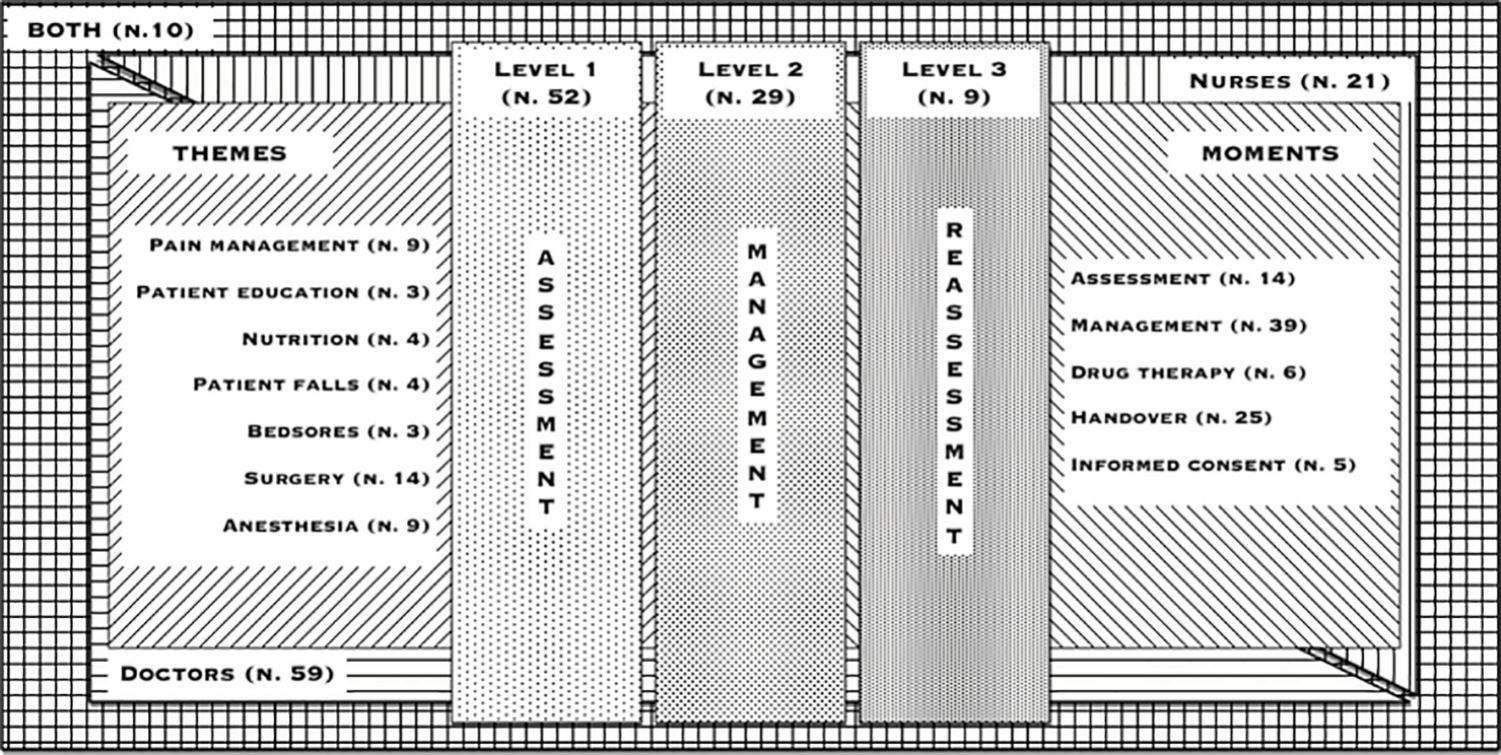
Model of the peer-review tool. The total number of items identified during the seven years experience reached a total of 92 items. The items belong to different healthcare workers profiles: doctors (n. 59 items), nurses (n. 21), both of them (n. 10) and therapist (n. 2 –not shown). The items, moreover, explore 7 themes (n. 46), 5 moments (n. 89) and 3 levels (n. 90).

All items identified during these years (2010–2016) reached a total of 92, and they assessed activities that belong to different HCWs profiles: doctors (59 items), nurses (21 items), both of them (10 items) and therapists (2 items). The items explored different themes and moments of the hospitalization, as well as multiple levels reported in the CR, as shown in **Fig 1**. The main motivation for categorizing items was to group them into homogeneous main subject of patient care (*themes*) and in different steps within hospitalization (*moments*), considering also the role responsible for the activities. Explored themes consisted of: *pain management* (9 items), *patient education* (3 items), *nutrition* (4 items), *patient falls* (4 items), *bedsores* (3 items), *surgery* (14 items) and *anaesthesia* (9 items). Moments were: *initial assessment* (14 items), *management* (39 items), *drug therapy* (6 items), *handover* (25 items) and *informed consent* (5 items). The peer-review electronic tool was designed to explore items also in multiple levels, which should be considered as progressive steps in the care process. *Level 1* examined documentation of relevant clinical assessments in CR (52 items)–for example, initial evaluation of pain. *Level 2* investigated documentation of an in-depth analysis regarding that specific topic (29 items)–for example, details on the pain location or response to drugs. *Level 3* evaluated re-assessment of the specific issue after intervention (9 items)–for example, pain evaluation in a specific time span after drug therapy. Achievement the third-level item was considered a marker of a healthcare system quality, as it demonstrates both measurement and management of the whole specific care process (in our example: correct and effective pain management). HCWs profiles, themes, moments and levels frameworks intersections within CR completeness peer-review tool are shown in **Fig 1**.

**Fig 2:**
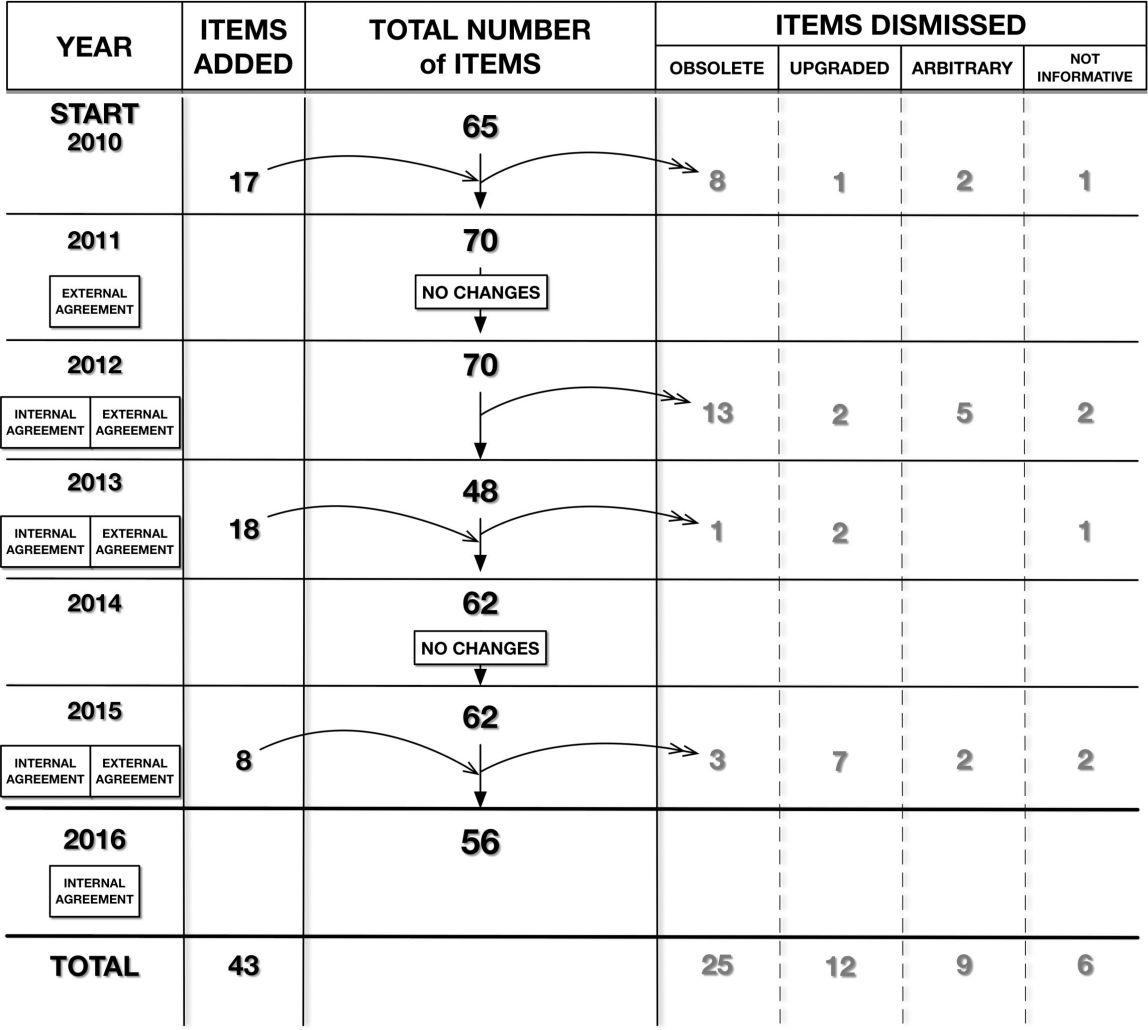
The evolution of items of the peer-review tool. The tool started in 2010 with 65 items and arrived at 56 items in 2016. During these years the items identified changed over time from a minimum of 59 (2013–2014) to a maximum of 70 (2011–2012). Some items were added (n. 43 items), some others were dismissed because considered obsolete (n. 25), arbitrary (n. 9), not informative (n. 6) or ready to be upgraded (n. 12) to better explore other phases of clinical care. No changes in the peer-review tool occurred in 2011 and 2014. Internal agreement was performed once a year from 2012 to 2016, while external agreement was performed from 2011 to 2015, both with the exception of 2014.

The tool changed over this period: the number of items varied annually from 65 at the beginning to 56 final ones, with some items being dismissed, other added, and still others upgraded to better explore more advanced phases of clinical care. Among the dismissed items: 25 were deemed obsolete, six not enough informative (due to the sporadic information assessed), nine too arbitrary and 12 ready for upgrade (≥95% compliant for three consecutive quarters, at hospital-aggregated level). Among the 43 items added: 35 were the update version of previous items, while eight were completely new (**Fig 2**). Examples of items stopped because they already had high levels of compliance were those investigating the surgical report and the discharge letter. Examples of too arbitrary items were those regarding readability of handwritten clinical notes. Items about participation in clinical trials and obtaining informed consent, which were too sporadic, were found to be insufficiently informative.

### Sample size and data collection

To the best of our knowledge, there are no published studies on the effectiveness of the peer-review tool for CR quality assurance in improving CR completeness. Therefore, in the absence of previous data and to estimate an appropriate sample size, we decided to sample quarterly a minimum of 300 CRs out of 7,500 discharges (rule of thumb). To be relevant for all units of the hospital we selected eight inpatient paper-based CRs for each of the 42 units in each trimester. Data were collected every three months by the appointed HCWs, transmitted to and processed by the Quality Unit (QU). Then the results obtained have been reported in two-weeks-time to every single unit and, after aggregation, to the hospital leadership. Items concerning therapists were taken into consideration only for the total CR completeness and not for the specific professional attitude because of insufficient observations.

According to the research policy at the Academic Hospital of Udine, this work was part of the quality improvement project concerning the improvement of clinical records, meeting the criteria for operational quality improvement activities exempt from ethics review (focused on process, functional and operational areas—not patients). Neither patient’s personal identifiers, nor clinical information were recorded, data were fully anonymized before being accessed by QU and authors of this paper, making tracing the CRs back impossible and thus not requiring formal ethical approval according to European regulation (EU-GDPR).

### Data validation

The QU established the tool and the data validation process, which consisted in two steps of agreement evaluation: internal and external agreement. Joint-probability of agreement is presented in this article, but no kappa-statistics or other reliability coefficients were calculated. The internal agreement was conducted by 17 experts of the QU, who blindly and independently assessed the same two CRs randomly selected, using the peer-review tool. The percentage of agreement between them was then calculated considering as the gold standard the review performed by the leader of QU. The main disagreements that arose during the review process were discussed in group and discrepancies were resolved. More detailed instructions on how to use the tool were then developed to standardize member assessments from QU and improve consistency and reliability. Internal agreement was performed once a year from 2012 to 2016, except for 2014, year in which no changes were made on the tool (**Fig 2**).

In the external agreement we blindly tested the degree (percentage) of agreement between the appointed HCWs (peer-reviewers) and the QU members, considered as gold standard. In the external agreement two CRs, randomly selected from those just reviewed by the appointed HCWs, were blindly assessed by professionals of the QU Unit. The proportion of agreement in reviewing between the results from the appointed HCWs and the QU members was then calculated with the purpose of assuring the quality of the data collected by appointed doctors and nurses, and therefore the reliability of the collected data. The main disagreements emerged during external agreement were discussed with peer-reviewers. In order to standardize future peer-reviews and improve agreement and reliability, more detailed instructions for tool application were developed and shared with the appointed HCWs. External agreement was performed from 2011 to 2015 after major changes of the tool items (new items added or items dismissed), major changes of CR and in case of new HCWs involved in the peer-review process (**Fig 2**).

### Data analysis

Variations in the proportions of CR completeness were evaluated through chi-square statistics (χ^2^) and chi-square tests for departure (χ^2^_d_) from linear trend (Cochran-Armitage test). An alpha-level of 0.05 was chosen as a guide for significance. Data analysis was performed using STATA (StataCorp. 2013. Stata Statistical Software: Release 13. College Station, TX: StataCorp LP).

## Results

**Fig 3:**
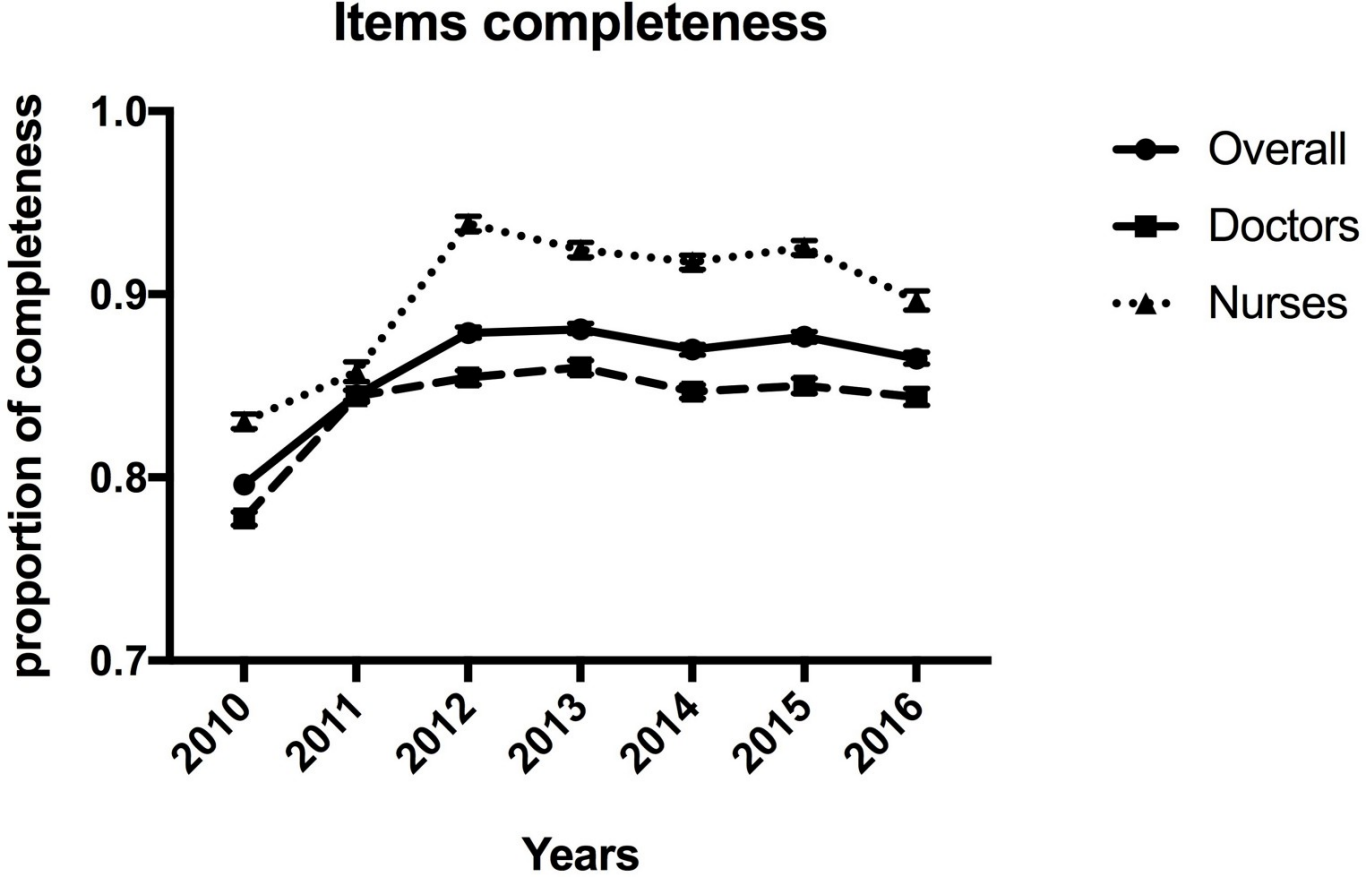
Overall and professional role proportion of items completeness. The overall completeness improved from 79.6% in 2010 reaching 88.1% in 2013. The completeness of doctors’ items reached a maximum of 86.3% in 2013, starting from 77.8% in 2010. Nurses’ items increase from 83.1% in 2010 to a maximum of 91.5% in 2013.

In the 2010–2016 period a total of 9,408 CRs (336 CRs quarterly) were evaluated by the doctors and nurses, trained as previously described, using the specific peer-review electronic tool. From 2010 to 2016 the overall completeness of CR improved significantly (χ^2^, p<0.001), notably in the early years when from 79.6% in 2010 it reached 88.1% in 2013, settling to 86.5% in 2016 with a not linear overall trend (χ^2^_d_, p<0.001) (**Fig 3**). As shown in **Fig 3**, analysing data about doctors and nurses separately, doctors’ behaviour showed a trend that is similar to the aggregated one [significant overall trend (χ^2^, p<0.001), although not linear (χ^2^_d_, p<0.001)]: from 77.8% in 2010 to a maximum of 86.0% in 2013, while nurses improved more consistently, reaching higher level of completeness: from 83.1% in 2010 to a maximum of 92.4% in 2013 (χ^2^, p<0.001; χ^2^_d_, p<0.001). Further details can be obtained consulting **Table 1.**

**Table 1:** Item completeness by professional role.

Role	Completeness of Clinical Records							Trend tests	
	2010	2011	2012	2013	2014	2015	2016	p(χ^2^)	p(χ^2^_d_)
	y/tot	y/tot	y/tot	y/tot	y/tot	y/tot	y/tot		
	(%)	(%)	(%)	(%)	(%)	(%)	(%)		
***Doctors***	41,236/53,035 (77.8)	40,664/48,162 (84.4)	27,874/32,619 (85.5)	27,130/31,541 (86.0)	29,101/34,363 (84.7)	25,536/30,042 (85.0)	20,124/23,844 (84.4)	<0.001	<0.001
***Nurses***	28,877/34,765 (83.1)	14,365/16,748 (85.8)	12,868/13,708 (93.9)	15,796/17,087 (92.4)	17,224/18,772 (91.8)	16,119/17,415 (92.6)	11,422/12,738 (89.7)	<0.001	<0.001
***Both***	1,082/1,602 (67.5)	911/1,294 (70.4)	962/1,109 (86.7)	1,270/1,515 (83.8)	1,366/1,696 (80.5)	1,226/1,454 (84.3)	3,246/3,668 (88.5)	<0.001	<0.001
**Total** *	71,195/89,402 (79.6)	55,940/66,204 (84.5)	41,704/47,436 (87.8)	44,196/50,143 (88.1)	47,980/55,167 (87.0)	43,147/49,211 (87.7)	35,345/40,857 (86.5)	<0.001	<0.001

Trend tests are divided by role: doctors, nurses, items shared by both of them and total. Results are divided by number of positive answers to the items, meaning completeness (y), total number of items analyzed (tot) and the proportion of completeness (%). Trend tests are shown as p-value for chi-squared for trend (χ^2^) and chi-squared for departure (χ^2^_d_).

*In Total are also included professionals as therapist, that are not specifically analyzed because of not enough observations.

All themes, except *patient falls*, registered a significant (χ^2^, p<0.001) improvement in first years, and then flattened in the last years (χ^2^_d_, p<0.001). Particularly, in the items investigating *nutrition*, doctors improved from 65.9% (2010) to 89.3% (2012) and nurses from 59.5% (2011) to 79.6% (2013). Regarding *bedsores* (χ^2^, p = 0.0121) nurses improved from 82.6% (2010) to 95.0% (2012). As far as *pain management* is concerned, doctors improved from 59.9% (2010) to 94.3% (2013). Moreover, considerable completeness level was achieved in *patient education*, reaching a maximum of 91.4% for doctors and 94.4% for nurses in 2016, with a significant overall trend, although not linear (χ^2^, p<0.001; χ^2^_d_, p<0.001).

Examining moments for both HCWs profiles, completeness of CR achieved 92.6% in *initial assessment* in 2016 (doctors: 88.6%, nurses: 96.2%) and a maximum of 90.2% in *management* in 2012 (doctors: 87.1%, nurses: 94.8%) with a significant (χ^2^, p<0.001) improvement, although not linear also in these cases (χ^2^_d_, p<0.001). Conversely the less compliant item was *drug therapy*, which reached a maximum of 80.0% completeness level in 2014, settling to 74.1% in 2016. More detailed information can be found in **Table 2**.

**Table 2:** Trend tests by themes and by moments and professional role.

Themes / Moments		Role	Completeness of Clinical Records							Trend tests	
			2010	2011	2012	2013	2014	2015	2016	p(χ^2^)	p(χ^2^_d_)
			y/tot	y/tot	y/tot	y/tot	y/tot	y/tot	y/tot		
			(%)	(%)	(%)	(%)	(%)	(%)	(%)		
***Themes***	***Anesthesia***	**D** *	1,461/1,683 (86.8)	1,546/1,830 (84.5)	1,430/1,588 (90.1)	1,540/1,876 (82.1)	1,576/1,980 (79.6)	1,318/1,652 (79.8)	820/1,049 (78.2)	<0.001	<0.001
	***Pain***		913/1.524 (59.9)	3,542/4,171 (84.9)	3,368/3,644 (92.4)	3,806/4,036 (94.3)	4,107/4,683 (87.7)	3,747/4,216 (88.9)	3,206/3,628 (88.4)	<0.001	<0.001
	***Surgery***		2,038/2,167 (94.0)	4,546/4,873 (93.3)	2,664/2,897 (92.0)	2,178/2,438 (89.3)	2,981/3,606 (82.7)	2,454/2,960 (82.9)	2,245/2,717 (82.6)	<0.001	<0.001
	***Falls***	**N** **	672/800 (84.0)	1,679/1,841 (91.2)	1,483/1,560 (95.1)	2,722/3,018 (90.2)	3,283/3,634 (90.3)	3,217/3,559 (90.4)	3,184/3,474 (91.5)	0.0844	<0.001
	***Bedsores***		642/777 (82.6)	1,596/1,797 (88.8)	1,414/1,488 (95.0)	1,978/2,234 (88.5)	2,273/2,575 (88.3)	2,050/2,285 (89.7)	2,050/2,261 (90.7)	0.0121	<0.001
	***Nutrition***	**D**	0/0 (-)	863/1,450 (59,5)	976/1,294 (75.4)	1,041/1,281 (79.6)	1,019/1,334 (76.4)	835/1,193 (70.0)	840/1,174 (71.6)	<0.001	<0.001
		**N**	531/806 (65.9)	1,370/1,784 (76.8)	1,369/1,533 (89.3)	1,710/1,969 (86.8)	1,871/2,267 (82.5)	1,811/2,149 (84.3)	1,699/2,009 (84.6)	<0.001	<0.001
	***Education***	**D**	559/753 (74.2)	915/1,209 (75.7)	939/1,113 (84.4)	1,000/1,169 (85.5)	1,033/1,163 (88.8)	931/1,019 (91.4)	958/1,048 (91.4)	<0.001	<0.001
		**N**	598/1,222 (48.9)	2,038/2,758 (73.9)	2,167/2,438 (88.9)	2,261/2,498 (90.5)	2,300/2,502 (91.9)	2,127/2,289 (92.9)	2,158/2,285 (94.4)	<0.001	<0.001
***Moments***	***Assessment***	**D**	3,436/4,550 (75.5)	7,197/8,368 (86.0)	5,572/6,556 (85.0)	5,240/5,867 (89.3)	5,268/5,875 (89.7)	4,690/5,218 (89.9)	3,498/3,948 (88.6)	<0.001	<0.001
		**N**	2,857/3,859 (74.0)	6,231/7,202 (86.5)	5,782/6,239 (92.7)	5,953/6,231 (95.5)	6,021/6,358 (94.7)	5.497/5,768 (95.3)	4,313/4,484 (96.2)	<0.001	<0.001
		**T**	6,293/8,409 (74.8)	13,428/15,570 (86.2)	11,354/12,795 (88.7)	11,193/12,098 (92.5)	11,289/12,233 (92.3)	10,187/10,986 (92.7)	7,811/8,432 (92.6)	<0.001	<0.001
	***Management***	**D**	6,186/8,610 (71.8)	8,307/10,554 (78.7)	8,262/9,486 (87.1)	10,614/12,451 (85.2)	11,553/13,973 (82.7)	10,249/12,188 (84.1)	9,448/10,951 (86.3)	<0.001	<0.001
		**N**	11,866/14,240 (83.3)	6,830/8,096 (84.4)	6,778/7,149 (94.8)	9,843/10,856 (90.7)	11,203/12,414 (90.2)	10,622/11,647 (91.2)	6,812/7,778 (87.6)	<0.001	<0.001
		**B**	1,082/1,602 (67.5)	911/1,294 (70.4)	962/1,109 (86.7)	1,270/1,515 (83.8)	1,366/1,696 (80.5)	1,226/1,454 (84.3)	2,406/2,635 (91.3)	<0.001	<0.001
		**T** ***	19,134/24,452 (78.3)	16,048/19,944 (80.5)	16.002/17,744 (90.2)	21,727/24,822 (87.5)	24,411/28,418 (85.9)	22,363/25,589 (87.4)	18,934/21,655 (87.4)	<0.001	<0.001
	***Drug therapy***	**D**	20,346/27,615 (73.7)	4,724/7,137 (66.2)	4,614/6,441 (71.6)	5,086/6,451 (78.8)	5,267/6,582 (80.0)	4,592/5,886 (78.0)	3,446/4,650 (74.1)	<0.001	<0.001
		**N**	14,154/16,666 (84.9)	1,304/1,450 (89.9)	308/320 (96.2)	0/0 (-)	0/0 (-)	0/0 (-)	0/0 (-)	<0.001	= 1.000
		**T**	34,500/44,281 (77.9)	6,028/8,587 (70.2)	4,922/6,761 (72.8)	5,086/6,451 (78.8)	5,267/6,582 (80.0)	4,592/5,886 (78.0)	3,446/4,650 (74.1)	0.4669	<0.001
	***Handover***	**D**	10,039/10,640 (94.4)	17,301/18,291 (94.6)	6,956/7,365 (94.4)	4,047/4,395 (92.1)	4,908/5,643 (87.0)	4,177/4,763 (87.7)	1,963/2,406 (81.6)	<0.001	<0.001
	***Informed consent***	**D**	1,229/1,602 (75.9)	3,135/3,812 (82.2)	2,470/2,781 (89.1)	2,143/2,377 (90.2)	2,105/2,290 (91.9)	1,828/1,987 (92.0)	1,769/1,889 (93.6)	<0.001	<0.001

D: doctors; N: nurses; B: both; T: total; ***

Themes are: anesthesia, pain, surgery, falls, bedsores, nutrition and education. Moments are: assessment, management, drug therapy, handover, informed consent. Results are divided by role: doctors (D), nurses (N), items shared by both of them (B) and total (T). Results are divided by number of positive answer to the items, meaning completeness (y), total number of items analyzed (tot) and the proportion of completeness (%). Trend tests are shown as p-value for chi-squared for trend (χ^2^) and chi-squared for departure (χ^2^_d_).

*only doctors; nurses: insufficient observations.

**only nurses; doctors: insufficient observations.

***Total in management (moment) also included professionals as therapist, that are not specifically analyzed because of not enough observations.

**Fig 4:**
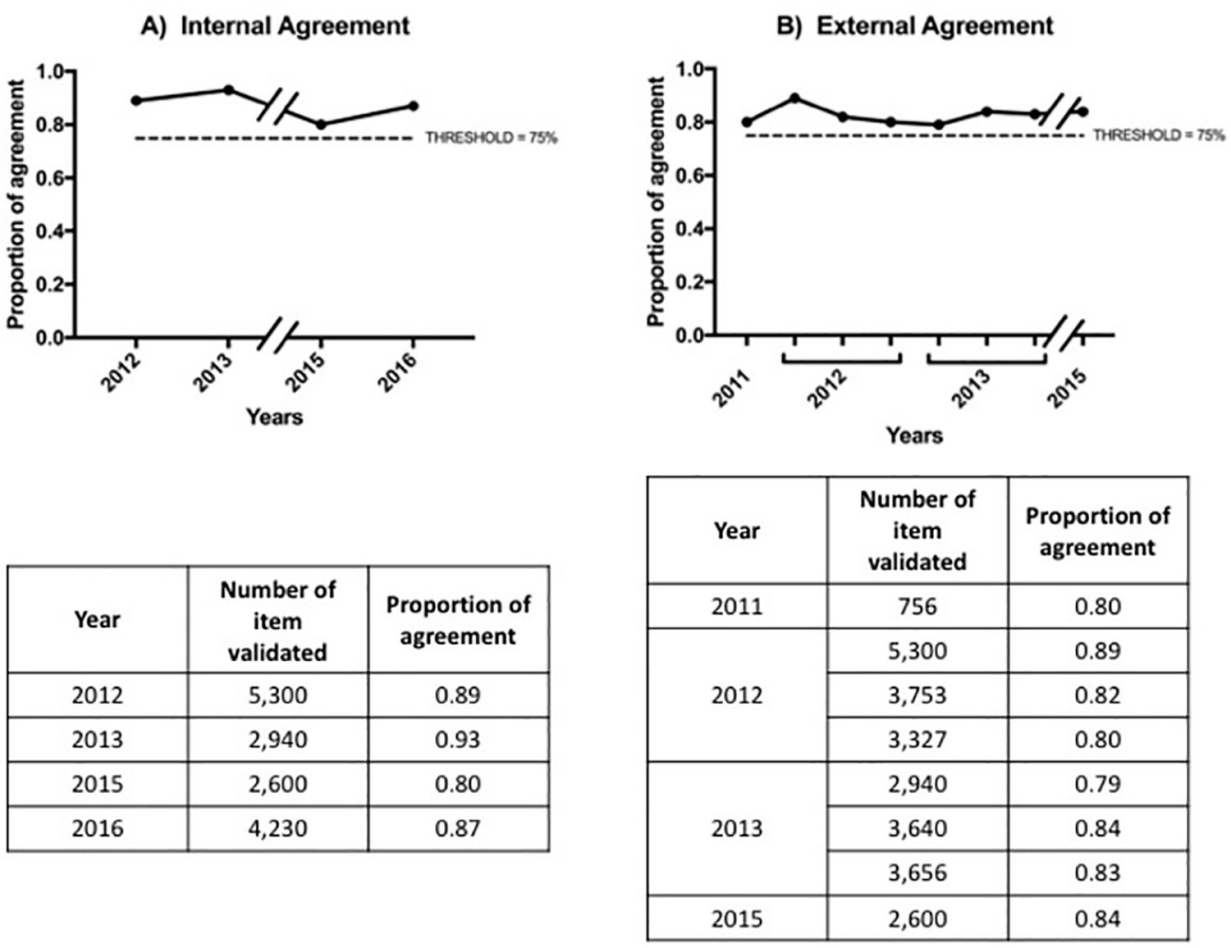
Proportion of internal (A) and external (B) agreement in the data validation process. Internal agreement was performed once a year from 2012 to 2016, with the exception of 2014. External agreement was performed from 2011 to 2015 after the major changes of items, major changes of CR and in case of new professionals involved. The two proportions of agreement were above the threshold (75%) decided by the Quality Unit as satisfactory. Table legends show, for each year, the proportion of agreement and the number of items validated by the professionals of the Quality Unit and the peer-reviewers from each units.

All the three levels achieved a significant improvement (χ^2^, p<0.001), although not linear (χ^2^_d_, p<0.001). Particularly, *Level 1*-assessment started in 2010 with 80.0%, reaching 89.4% in 2016, remained at a higher completeness than *Levels 2*-management (82.9%), and *Level 3*-re-assessment (82.4%), as shown in **Table 3**. When analysing data according to levels framework, overall result is driven up by nurses’ items completeness: for *Level 1*, in fact, nurses achieved the completeness of 96.6% as a maximum in 2015, while doctors stopped at 88.4% in 2014. Results of both levels of data validation (internal and external agreements) were always above the proportion of 75%. This cut-off was selected by the hospital leadership as satisfactory for assuring the quality of data collected and so the reliability of the performance measurement (**Fig 4**).

**Table 3:** Trend tests by levels.

Levels	Completeness of Clinical Records							Trend tests	
	2010	2011	2012	2013	2014	2015	2016	p(χ^2^)	p(χ^2^_d_)
	y/tot	y/tot	y/tot	y/tot	y/tot	y/tot	y/tot		
	(%)	(%)	(%)	(%)	(%)	(%)	(%)		
***Level 1***	6,8964/8,6228 (80.0)	43940/50800 (86.5)	31469/35542 (88.5)	29012/31862 (91.1)	29730/32723 (90.9)	26453/29083 (91.0)	20741/23190 (89.4)	<0.001	<0.001
***Level 2***	1845/2632 (75.9)	9442/11869 (79.6)	7556/8785 (86.0)	8919/10641 (83.8)	10794/12132 (82.2)	9626/11388 (84.5)	8054/9719 (82.9)	<0.001	<0.001
***Level 3***	386/742 (52.0)	2558/3535 (72.4)	2679/3109 (86.2)	6265/7640 (82.0)	7456/9312 (80.1)	7068/8740 (80.9)	6550/7948 (82.4)	<0.001	<0.001

Results are divided by number of positive answer to the items, meaning completeness (y), total number of items analyzed (tot) and the proportion of completeness (%). Trend tests are shown as p-value for chi-squared for trend (χ^2^) and chi-squared for departure (χ^2^_d_).

## Discussion

Clinical record is the main communication tool used by HCWs to ensure safe continuity of care. As primary source of written evidence of the care provided to patients, CR is the best source in order to evaluate quality and appropriateness of care in clinical governance [6]. With this study we answered the need to evaluate the information recorded by professionals in CRs and ensure that accurate and complete data are recorded consistently [6, 14, 15, 26].

The completeness of CR, assessed using this peer-review tool, showed an overall positive and significant trend through these seven years of experience, especially in early years. Although doctors’ diligence showed a positive trend that was similar to the overall assessment, nurses improved more consistently.

Except for the first two years, our findings showed an overall completeness of CR always being above 85% and demonstrated an improvement through the seven years of this experience. Level of completeness in the 42 Units remained above the threshold, even though some items have been changed over years, with old items becoming obsolete and new items being added for a deeper analysis of the care process, as required by the QU. Our results, therefore, reflect a good quality practice [21], especially in some clinical areas and moments of care. Both themes and moments, in fact, registered significant improvements, indicating greater attention by HCWs in several clinical areas, such as *patient education* and *bedsores*, or in different periods of care, as for *initial assessment* and *management*. Concerning moments, however, *drug therapy* only registered a slight improvement. This is probably due to the complexity of the explored *drug therapy* items. For example, the item concerning prescription aggregated several fields included in CR (legibility, indication of active substance, dose and route of administration, date and hour of prescription, medical doctor’s signature). Therefore, it was more challenging for professionals to fill in all requested fields simultaneously. However, the reduced completeness in *drug therapy* items could be a predictor of poor quality of care [10, 11, 23]. These findings are consistent with prior research [12, 18] and suggested that further investments and attention should be put on doctors behaviour in order to prevent potentially harmful consequences for patient safety [11, 12, 23]. Despite the results obtained in themes and moments, there is the opportunity to improve performance as far as levels are concerned, achieving a better completeness in the *Level 2*-management and *Level 3*-re-assessment, pursuing a better quality of care. As the initial assessment reflects the first approach to the patient, the higher completeness of *Level 1* items can be explained if we assume that professionals were already used to record it from the beginning. On the contrary, management (*Level 2*) and re-assessment (*Level 3*) represent progressive levels of care and registered slight improvements in completeness probably because of the greater difficulty to implement them. The multilevel evolution of items, indeed, reflects the growth of both quality of care provided and its documentation in clinical records by HCWs.

Furthermore, data obtained in this 7-year experience point out different diligence between professional profiles, as doctors practice showed a positive trend, even though nurses improved more consistently. These data may suggest a recalcitrant attitude among physicians, whereas nurses appear to be more diligent in following procedures and document actions, as we previously demonstrated for Incident Reporting [33] and for drug prescription [18]. Our findings confirm existing literature on evaluating peer-review and feedbacks processes, which suggest that physicians are relatively poor at self-assessment [34], probably because they tend to seek continuing improvement in their specific areas of interest, rather than areas of greatest and common need [35]. These findings should be taken into account for further development of both the feedback method and this tool, such as exploring more in detail doctors items, in particular *drug therapy [18]* and *nutrition [29, 36]*.

This electronic peer-review tool was structured to provide consistent measurement and being unobtrusive while respecting HCWs independence at the same time, as suggested by *Wang et al*. [37]. Moreover, the combination of peer-review by skilled HCWs and the possibility to confirm data with external agreement allowed the QU and the hospital leadership to obtain a reproducible and reliable picture of CR completeness within the Hospital. The transparency of data and a clear information flow is also fundamental for the hospital leadership in order to receive the message regarding performance as intended [35]. The involvement of appointed HCWs from each unit, in addition, enabled the hospital QU to collect a large amount of data in short time, as well as to give short-term feedback to both units and the hospital leadership. In this way we think to have optimised the feedback process with a relatively inexpensive strategy, as wished by *Ivers et al*. [35, 38–41]. These short-term feedbacks, given to professionals in two-weeks-time, enabled every unit to discuss its own performance during internal periodic meetings, and to implement actions or interventions for continuous improvement purposes. The whole review and feedback process enable the healthcare organization to reach improvements in a three-months-period and to keep monitoring CR completeness. The effectiveness of this process is supported also by the long-term follow-on provided by the QU. Our two-way information flow (from HCWs to Quality Unit and back) confirm findings of literature about the efficacy of rapid analysis of data, intensive feedback, and data delivering by representative and respected colleagues [30].

Although the CRs adopted in the Academic Hospital of Udine were paper-based, we are aware that several authors exhorted the adoption of electronic health records [42, 43]. Since recording information in CR is burdensome and time-consuming, electronic health records may contribute to facilitate the recording, supporting completeness, accessibility and exchange of patient information [4, 44]. Although data of electronic clinical records are entered by humans and therefore prone to error, they might be more complete, given the possibility of integrating multiple data sources [45]. The tool and the process of feedback we implemented is compatible with these systems: it can be facilitated in collecting health and social data. In addition, previous literature demonstrated the effectiveness of audit and feedback to clinicians for quality improvement initiatives using real-time data generation from electronic health records [46–49], allowing a more detailed examination of quality of care and operational inefficiencies [42].

Although several articles address CR completeness, they are characterized by diversity in the population observed and in extracted elements examined from CR [7, 12–14, 18, 26, 31, 50–52]. Our results derive from all CR sections as a whole and from all hospital units. We deem this information characteristic a strength, because it allowed the QU to collect a large amount of data from both clinical and surgical departments, but also from highly-specialized units as neurosurgery, hearth surgery, pediatrics and neonatal intensive care unit, solid organ transplant center and adult intensive care unit, while monitoring the whole hospital, after aggregation of data.

This peer-review method faces some limitations. First of all, data consistency could be difficult to be guaranteed, due to many HCWs belonging to different hospital units involved in the review. The large number of HCWs, in fact, requested a considerable educational effort in order to maintain tool knowledge updated when items or HCWs in charge of evaluation changed. Even if internal agreement missed in 2011 and 2014, and external agreement missed in 2014, since no major changes in items or appointed HCWs occurred in these years, we believe that missing data did not affect the peer-review process reliability, as both internal and external agreement were always over 75%. Moreover, although we performed the data validation through internal and external agreement (see Method section), we didn’t calculate any test score for reliability coefficient, such as kappa-statistics, and the reliability of collected data were evaluated only by joint-probability of agreement. Secondly, a diminished completeness of CR could be difficult to be distinguished from actual deficiency in clinical performance. Nevertheless, as several other authors [3, 12–16], we consider completeness CR as a proxy indicator of the quality of care provided to patients. Lack of CR completeness could be seen as an alert indicating that specific parts of CR are aimless or repetitive. Therefore, involving HCWs in the peer-review and feedback process could also be useful in order to modify the structure and usability of the CR, improving professional diligence toward recording medical information in it. In our opinion, such a new quality and clinical integrated approach could also benefit from a more active physicians involvement, because of their specific attention to clinical outcomes [31, 53].

Despite some limitations, the implementation of this periodical peer-review tool have several advantages and strengths. It is an unobtrusive and economical assessment tool, more reliable than non-quantitative methods, such as focus groups or clinical audits. The tool is structured with items familiar to HCWs and this facilitates data collection in two weeks-time. The improvement of the HCWs’ diligence in the registration of patient information are desirable to enhance quality of care, and thus patient safety and patient outcome. Peer-review [29], as well as feedbacks [30], are widely used strategies to promote improvement and induce desired changes [31]. Our approach allows HCWs to be part of the quality program, improving quality and safety of care by strengthening a safety climate, as wished by several studies, commentaries or analysis [9, 25, 28, 29]. Therefore, in our opinion, healthcare leadership should motivate healthcare professionals to participate in peer-review processes as an opportunity to look at their work and to be offered helpful criticism [29], in order to achieve safer practices, reducing medical errors and ensure patient safety. Although this tool has the limit of having been developed on and used with paper CR, measuring the HCWs’ behaviours and spreading the culture of healthcare quality allowed the Academic Hospital to deepen the level of process analysis and to highlight different compliance habits among HCWs profiles.

## Conclusions

This peer-review tool, structured to be unobtrusive and including representative items, enables the Quality Unit and leadership of the hospital to have a reliable picture of clinical records completeness as an indicator of quality of care. The adoption of this tool allows healthcare workers to be part of the quality process during evaluation, supporting them in self-improvement and in finding standards for clinical records keeping. This would benefit in improve completeness and accuracy of information, and thus might improve communications and patient outcome.
